# Effects of work-directed interventions on return-to-work in people on sick-leave for to common mental disorders—a systematic review

**DOI:** 10.1007/s00420-024-02068-w

**Published:** 2024-05-06

**Authors:** Elisabeth Brämberg, Elizabeth Åhsberg, Gunilla Fahlström, Elisabet Furberg, Carl Gornitzki, Anna Ringborg, Peter Skogman Thoursie

**Affiliations:** 1 Unit of Intervention- and Implementation Research for Worker Health, Institute of Environmental Medicine, Karolinska Institutet, 171 77 Stockholm, Sweden; 2 School of Public Health and Community Medicine, Institute of Medicine, Sahlgrenska Academy, University of Gothenburg, Gothenburg, Sweden; 3 Swedish Agency for Health Technology Assessment and Assessment of Social Services (SBU), Stockholm, Sweden; 4 Department of Public Health and Caring Sciences, Uppsala University, Uppsala, Sweden; 5 Department of Philosophy, Stockholm University, Stockholm, Sweden; 6 Department of Economics, Stockholm University, Stockholm, Sweden

**Keywords:** Systematic review, Depression, Anxiety, Adjustment disorder, Reactions to severe stress, Return-to-work

## Abstract

**Purpose:**

To evaluate the body of evidence of the effects of work-directed interventions on return-to-work for people on sick leave due to common mental disorders (i.e., mild to moderate depression, anxiety, adjustment disorders and reactions to severe stress).

**Methods:**

The systematic review was conducted in accordance with an a priori developed and registered protocol (Prospero CRD42021235586). The certainty of evidence was assessed by two independent reviewers using the Grading of Recommendations, Assessment, Development and Evaluations.

**Results:**

We reviewed 14,794 records published between 2015 and 2021. Of these, eight RCTs published in eleven articles were included in the analysis. Population: Working age adults (18 to 64 years), on sick leave due to mild to moderate depression, anxiety, adjustment disorders or reactions to severe stress. Intervention: Work-directed interventions. Comparator: No comparator, Standard care, or other measures. Outcome: return to work, number of days on sick leave, income. Overall, the effects of work-focused CBT and work-focused team-based support on RTW resulted in increased or faster return-to-work compared with standard care or no intervention (low certainty of evidence). The effects of Individual Placement and Support showed no difference in RTW compared with standard care (very low certainty of evidence).

**Conclusion:**

Interventions involving the workplace could increase the probability of RTW. Areas in need of improvement in the included studies, for example methodological issues, are discussed. Further, suggestions are made for improving methodological rigor when conducting large scale trials.

Common mental disorders (CMDs) incorporate depression, anxiety and adjustment disorders (Fisker et al. 2022). These conditions affect about one in six people of working age and are a major cause of absence from work (OECD 2021). CMDs affect the individual not only in terms of suffering and the risk of social isolation, but also potential reduction in income. In medium- and high-income countries, the diagnosis of depression is associated with the highest societal burden due to disability, decreased ability to work and years lost due to premature death (European Agency for Safety and Health at Work (EU-OSHA)). The likelihood of absence from work due to sickness is greater with respect to mental health problems, such as CMDs, than with physical health issues (Bryan et al. 2021). It is widely acknowledged that having paid employment offers significant health advantages (Marmot 2017; Modini et al. 2016; Schuring et al. 2017; van der Noordt et al. 2014). Consequently, it is crucial to implement effective interventions to support employees returning to work after sick leave.

At first glance, psychological and/or pharmacological treatment for CMDs would appear to be adequate interventions to reduce specific symptoms and decrease the duration of sick leave. However, these interventions have only a marginal effect on the duration of sick leave, return-to-work and other work-related outcomes (Nieuwenhuijsen et al. 2020). As an alternative, involving the work-directed measures in the return-to-work process is a commonly suggested measure for improving return-to-work rates (OECD 2021). To meet the rehabilitation needs of employees on sick leave due to depression, anxiety, adjustment disorder (Arends et al. 2012; Hogg et al. 2021; Nieuwenhuijsen et al. 2020), mental conditions (Dewa et al. 2015; Fadyl et al. 2020), common mental disorders (Mikkelsen and Rosholm 2018; Nigatu et al. 2016; Salomonsson et al. 2018), or a combination of mental or musculoskeletal conditions (Finnes et al. 2019a; van Vilsteren et al. 2015) several systematic reviews have evaluated the effectiveness of interventions involving work-directed measures, with reference to different outcomes.

Psychological interventions (e.g., cognitive behavioral therapy, CBT) are reported to have a small but significant effect in reducing sick leave (Finnes et al. 2019a), and a reduction in symptoms (Hogg et al. 2021; Salomonsson et al. 2018). Further, work-directed problem-solving interventions (based on CBT-principles) have shown a reduction in sick leave and increases in RTW outcomes (Arends et al. 2012; Dewa et al. 2015). A combination of workplace-, work-directed and clinical interventions has been evaluated in relation to sick leave, RTW and time elapsing until RTW (Nieuwenhuijsen et al. 2020; Nigatu et al. 2016; van Vilsteren et al. 2015) with minor effects on absence due to sickness and RTW. To summarize, these interventions seem to have the potential to reduce the length of sick leave, and increase time to return-to-work. Still, at the 12 months follow-up or longer, the interventions are not more effective compared to the control conditions.

The current paper reports the systematic review of work-directed interventions, i.e., interventions involving several stakeholders (health care, employer) and the delivery of the intervention in direct contact with the employer or a representative of the employer (e.g., the employee’s supervisor, human resources representative or occupational health services) (Carroll et al. 2010). These interventions commonly aim to support employees on sickness absence by focusing on temporarily modification of work tasks, to overcome barriers for work participation, as well as decreasing symptoms, work disability, strengthen workability or work-related self-efficacy (Nieuwenhuijsen et al. 2020).

As discussed above, previous systematic reviews have evaluated the effects in relation to work-related outcomes, revealing incongruent results. In this systematic review we have evaluated the effect of work-directed interventions (Carroll et al. 2010) aimed to support employees on sickness absence by focusing on temporary modification of work tasks, to overcome barriers to work participation, as well as reducing symptoms, work disability, strengthening workability or work-related self-efficacy (Nieuwenhuijsen et al. 2020).

Hence, we addressed recent studies on work-directed interventions, with a broad range of work-directed interventions, including general labor market programs. In addition to RCTs, the presented review allowed for quasi-experiments. Further, our systematic review contributes to the knowledge base by providing an analysis of ethical aspects arising when introducing work-directed interventions involving the employee, health care and workplace.

## Aim

To evaluate the body of evidence of the effects of work-directed interventions on return-to-work for people on sick leave due to common mental disorders (i.e., mild to moderate depression, anxiety, adjustment disorders and reactions to severe stress).

## Methods

This systematic review was conducted in accordance with the Preferred Reporting Items for Systematic Reviews and Meta-Analyses (PRISMA) 2020 statement (Page et al. 2021) and the Swedish Agency for Health Technology Assessment and Assessment of Social Services Method (sbu.se).

### Protocol and registration

The systematic review was conducted in accordance with an a priori developed and registered protocol Prospero CRD42021235586 https://www.crd.york.ac.uk/prospero/display_record.php?ID=CRD42021235586. No deviations from the protocol were made.

### Eligibility criteria

Randomized controlled trials, cluster-randomized controlled trials, quasi-experimental observation studies and qualitative studies were included, provided they met the following criteria:

### Inclusion criteria

#### Population

Working age adults (18 to 64 years), on sick leave due to mild to moderate depression, anxiety, adjustment disorders or reactions to severe stress.

#### Intervention

Work-directed interventions are defined as involving several stakeholders including at least health care services, the employer and the person on sick leave/employee and delivered in direct contact with the employer or a representative of the employer.

#### Comparator

No comparator, standard care or other measures.

#### Outcome

Primary: return to work, number of days on sick leave, and income. Secondary: health measures (sleep, depression, anxiety, stress, quality of life, capacity for work) and the experience of participating in work-directed interventions.

#### Language

English, Swedish, Norwegian and Danish.

#### Publication type

Original reports published in peer-reviewed journals, or elsewhere (so called ‘grey literature’).

#### Search period

1995 to 2021. The final search was conducted in February, 2022.

#### Exclusion criteria

Studies including newly arrived immigrants, post-traumatic stress disorder or severe mental illness were excluded. Studies reporting experiences of sick-leave were also excluded.

### Search strategy and information sources

A systematic literature search was conducted in collaboration with an information specialist (CG). The following electronic databases were searched from 1995 to 2021 and the final search was conducted on February, 2, 2022: Medline (Ovid), Scopus (Elsevier), Ebsco Multi-Search (Psychology and Behavioral Sciences Collection; SocINDEX with Full Text; Academic Search Premier, ERIC); APA Psychinfo (Ebsco); Sociological Abstracts (ProQuest). The search terms were developed and defined in collaboration with the information specialist, and the authors (EBB, GF, EÅ, PST). The full list of search terms is presented in appendix 1. Additional searches of systematic reviews, health technology assessments reports and Swedish reports (‘grey literature’) were undertaken in Epistemonikos; International HTA Database, and KSR Evidence. The reference lists of included articles were searched, to identify additional studies. Articles included in systematic reviews were also checked for eligibility.

### Selection process

Initially, two reviewers (EÅ,GF) independently assessed all retrieved records for relevance, by screening the title/abstract and excluding those not meeting the inclusion criteria. If in doubt, the record/abstract was included. Thereafter, two reviewers (EBB and PST) reviewed the abstracts for relevance. The assessments were registered in Rayyan https://www.rayyan.ai/. The potentially relevant articles identified by at least one of the reviewers were retrieved in full-text and their eligibility was assessed in terms of correspondence between the population, intervention, control, and outcome (i.e., PICO). All disagreements were discussed and resolved, if necessary, together with a third reviewer.

### Risk of bias assessment

The risk of bias (RoB) in each of the articles was assessed by two reviewers (EBB and PST) using the Cochrane RoB 2 tool (Sterne et al. 2019). Initially, the reviewers met and together reviewed 10 full text articles in order to calibrate their assessments. Thereafter, the reviewers assessed RoB independently. Reporting bias was not assessed because pre-published protocols reporting the design etc. of included studies were not available in all cases. Any disagreements were discussed and resolved by the reviewers together with a third reviewer if necessary. Articles with a low or a moderate risk of bias were included.

### Effect measures

The fixed effects model was used in the meta-analysis, using Review Manager version 5.4. For binary outcomes, odds ratios, Cohens d and hazard ratios were reported. For continuous outcomes, the mean difference (MD) was used, while the Standardized Mean Difference (SMD) was applied if different instruments had been used to measure an outcome.

### Synthesis methods

The included articles were summarized and described according to the participants’ characteristics, interventions, follow-up and the outcomes measured. Meta-analysis was conducted if the included articles were reasonably consistent, and the results adequately reported. The articles were categorized according to intervention and assigned to identical intervention categories. This resulted in three categories of intervention. When an outcome was reported by only one article, we conducted a synthesis without meta-analysis reporting MDs, effect sizes or hazard ratios.

### Assessing the certainty of the evidence

The certainty of evidence was assessed by two independent reviewers, using the Grading of Recommendations, Assessment, Development and Evaluations (GRADE) (Balshem et al. 2011). The syntheses are shown in tables for Summary of Findings for each intervention type, presenting the total number of participants, the effects per study, certainty of evidence followed by reason/s for downgrading and comments on each outcome.

## Results

**Fig. 1:**
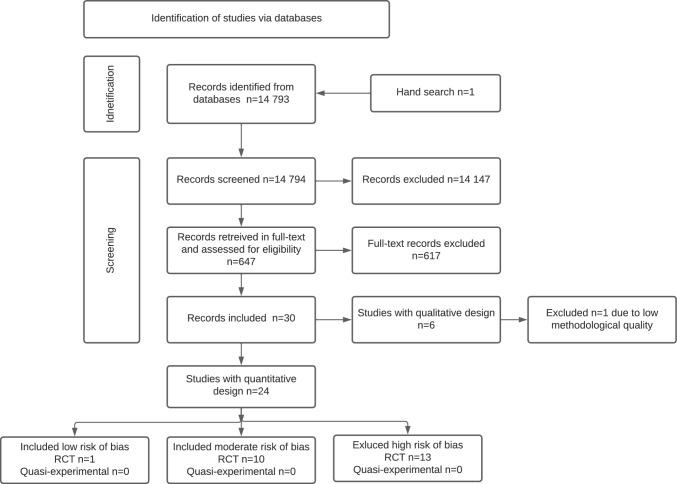
Flowchart over the study search and selection process

The literature search resulted in 14,794 records (see Fig. 1 for a flowchart), of which eight RCTs, published in eleven articles, were included (Bejerholm et al. 2017; Dalgaard et al. 2017a; Dalgaard et al. 2017b; Finnes et al. 2019b; Hellström et al. 2017; Hoff et al. 2022; Lammerts et al. 2016; Overland et al. 2018; Reme et al. 2015; Salomonsson et al. 2017; Salomonsson et al. 2020). Three studies were conducted in Sweden, three in Denmark, one in Norway and one in The Netherlands, all published between 2015 and 2021. The number of participants included in the studies varied between 61 and 1193, with a total of 2902, whom about 70% were female. The participants (median age between 34 and 46 years) were on part- or full-time sick leave. No adverse events were reported with respect to the interventions. The study characteristics are presented in Table 1, the interventions and comparisons in Table 2 and appendix 3 and 4. Reasons for exclusion are presented in appendix 2.

**Table 1:** Characteristics of the included randomized controlled trials

Study country	Study design Total N randomized	Mean age (SD and/or range), age categories N (%) female gender	Highest level of education	Intervention classification	Name of intervention Name of control condition Intervention (I) Control (C)	Longest follow-up	Outcomes
(Bejerholm et al. 2017) Sweden	RCT 63	41 (mean only) 44 (72)	University (31%)	Individual placement and support	I: Individual Enabling Support C: Traditional vocational rehabilitation	12 months	Employment rate Internship Education Pre-vocational activity/training
(Dalgaard et al. 2017a) Denmark	RCT 163	Intervention 45 years (range 28–60) Control group A 44 (range 29–63) Control group B 46 (range 26–62) 115 (70)	Higher education (years) Long (> 4) 9%	Work-focused behavioural therapy	I: Work-focused cognitive behavioural intervention C: No intervention or clinical assessment	10 months	Perceived stress (Perceived stress questionnaire) General Health (GHQ-12)
(Dalgaard et al. 2017b) Denmark	RCT 163	Intervention 45 (range 28 – 60) Control group A 44 (29 – 63) Control group B 46 (26 – 62) 115 (70)	Higher education (years) Long (> 4) 17%	Work-focused behavioural therapy	I: Work-focused cognitive behavioural intervention C: No intervention or clinical assessment	44 weeks	Lasting return to work Time until lasting return to work Stress Sleep
(Finnes et al. 2019b) Sweden	RCT 352	46.3 (8.89, range 22–61) 276 (78.4%)	University 205 (59.1)	Work-focused behavioural therapy	I: Acceptance and commitment therapy I: Workplace Dialogue Intervention I: Acceptance and commitment therapy and Workplace Dialogue Intervention C: Treatment as usual	9 months	Sickness absence net days Work functioning
(Hellström et al. 2017) Denmark	RCT 326	Intervention 34 (10) Control 36 (11) 221 (67.8)	University 30 (9.2)	Individual placement and support	I: Individual placement and support modified for people with mood or anxiety disorder C: Service as usual	24 months	Competitive employment or education
(Hoff et al. 2022) Denmark	RCT 666	Intervention (INT) 45.22 (9.42) Intervention (MHC) 45.26 (10.08) Control 43.18 (10.23 487 (73)	Bachelor or above 396 (59)	Work-focused team-based support	I: Integrated intervention C: Mental health care	12 months	Time to stable RTW
(Lammerts et al. 2016) The Netherlands	RCT 186	45.7 (10.6) 92 (49.5)	Low educational level (no education, primary school or lower vocational education) 49 (26.3)	Work-focused team-based support	I: The return-to-work programme C: Standard occupational healthcare	12 months	Duration in calendar days from day of enrolment in the study until first paid employment in a regular work-setting for ≥ 28 consecutive calendar days
(Overland et al. 2018) Norway	RCT 1193	40.4 802 (67.2)	University 657 (55)	Work-focused behavioural therapy	I: At Work and Coping C: Standard care	46 months	Work no benefits (i.e. employment or income and no benefits during a calendar month)
(Reme et al. 2015) Norway	RCT 1193	40.4 802 (67.2)	University 657 (55)	Work-focused behavioural therapy	I: At Work and Coping C: Standard care	12 months	Increased or maintained work participation Anxiety Depression Quality of life
(Salomonsson et al. 2017) Sweden	RCT 211	Cognitive behavioural therapy 42.5 (9.2, range 23–62) Return-to-work intervention 174 (82.4) Combination treatment 41.5 (10.4, range 22–64)	College/university ≥ 4 yrs Cognitive behavioural therapy 23 (10.9) Return-to-work intervention 31 (14.7) Combination treatment 27 (12.8)	Work-focused behavioural therapy	I: Cognitive behavioural therapy I: Return-to-work intervention I: Combination treatment	12 months	Net days of sick leave Psychiatric symptoms (Clinician Severity Rating) Depression Anxiety Perceived Stress Quality of life Work ability
(Salomonsson et al. 2020) Sweden	RCT 211	Cognitive behaviour therapy 42.0 (9.9) Return to work intervention 43.4 (9.3) Combination treatment 42.8 (9.9) 132 62.5)		Work-focused behavioural therapy	I: Cognitive behavioural therapy I: Return-to-work intervention I: Combination treatment	12 months	Net days of sick leave Psychiatric symptoms (Clinician Severity Rating) Symptoms of stress and exhaustion

**Table 2:** Description of interventions and comparisons

Study country	Intervention (type, content, frequency/duration)	Comparison	Comment
(Bejerholm et al. 2017) Sweden	Individual Enabling Support (IES). An employment specialist worked closely with the participant in relation to the outpatient team, family, Social Insurance Agency, Public Employment Service, and employers. One-hour weekly meetings until RTW. After RTW 20 min weekly meetings. Most IES principles corresponded to the IPS model	Traditional Vocational Rehabilitation (TVR) delivered by various professionals. The first step involves reducing symptoms and increasing work ability at a mental health service (1 h per week). Step 2 involves assessment of 50% work capacity (10–20 h per week). If work capacity is not met, the participant is encouraged to enter Step 3 with pre-vocational activities at the municipality, 5–20 h per week. The last step is vocational training during internship placements (20–40 h per week), and these can lead to employment positions	–
(Dalgaard et al. 2017a) Denmark	Work-focused cognitive behavioral therapy (CBT) consisted of six, one-hour sessions with individual work-focused CBT conducted by a psychologist over 16 weeks and an optional workplace intervention	*Control group A* Clinical assessment *Control group B* No intervention or clinical assessment	Randomization to control group B were closed prior to randomization to the intervention- and control group A because of a higher proportion than anticipated were excluded, based on the results of the clinical assessment (e.g. the participants’ stress condition was not adequately work-related)
(Dalgaard et al. 2017b) Denmark	Work-focused cognitive behavioral therapy (CBT) consisted of six, one-hour sessions with individual work-focused CBT conducted by a psychologist over 16 weeks and an optional workplace intervention	*Control group A* Clinical assessment *Control group B* No intervention or clinical assessment	See (Dalgaard et al. 2017a)
(Finnes et al. 2019b) Sweden	Acceptance and commitment therapy (ACT) The ACT protocol consisted of six manual-based face-to-face sessions based on the six core processes in the ACT-model: acceptance, mindfulness, defusion, self as context, values, and committed action Workplace Dialogue Intervention (WDI) The WDI aims at the facilitation of dialogue between the participant and the workplace through a series of steps involving the participant and the nearest supervisor. The aim is to generate mutual understanding on which arrangements are necessary or helpful in facilitating RTW ACT + WDI In the combined ACT and WDI condition, the two interventions as described were combined, resulting in nine intervention meetings. Two different therapists carried out the two interventions	Treatment as usual or rehabilitation in standard care facilities	–
(Hellström et al. 2017) Denmark	Individual Placement and Support modified for people recently diagnosed with mood or anxiety disorder (IPS-MA) included sessions 1–1,5 h/week, continued for as long as needed IPS is based on eight principles: eligibility based on client choice, focus on competitive employment, integration of mental health and employment services, attention to client preferences, work incentives planning, rapid job search, systematic job development and individualised job supports	Service as usual offered by Danish job centers, e.g. courses, company internship programs, wage subsidy jobs, skill development and guidance, mentor support or gradual return to employment. Normally, benefits can be received for a maximum of 52 weeks	–
(Hoff et al. 2022) Denmark	Integrated vocational rehabilitation and mental health care, in addition to service as usual containing teamsupport from municipal jobcenters and a mental healthcare team. A joint team from municipal jobcentres and mental healthcare gave service as usual In addition, closer support was given by an employment consultant (m = < 6 physical meetings, m = < 4 digital contacts) and care manager (m = < 22 weeks). A joint plan was formed with the participant. The support consisted of mentoring during job interviews, problem solving and how to manage job and illness in return to work	Service as usual included mental health care delivered by or via the participant’s general practitioner, private psychologist or psychiatrist. Further, vocational rehabilitation provided by Danish municipal job centers with management of the sickness benefit case, assessment of workability, and miscellaneous short-term programs with instruction and support for job searching. Job centers also offered unpaid internships and graded RTW depending on initial employment status	–
(Lammerts et al. 2017) The Netherlands	Participatory supportive RTW program in addition to usual occupational healthcare provided by a team of professionals, consisting of a RTW coordinator, an insurance physician, and a labor expert The intervention included a standardized form of occupational healthcare starting early after sick-listing (i.e. two weeks after allocation), with a participatory approach, integrated care and direct placement in a competitive job. The intervention started with analysis by the return-to-work coordinator and an analysis of medical problems by the insurance physician Within two weeks the participant meets with the coordinator and labor expert. Within another four weeks a vocational rehabilitation agency offers at least 2 competetive jobs	Usual occupational healthcare provided by a team of professionals, consisting of a return-to-work coordinator, an insurance physician, and a labor expert	–
(Overland et al. 2018) Norway	At Work and Coping Work-directed, individual cognitive behavioural therapy (CBT) combined with job support, delivered by teams of therapists and employment specialists at each site. Up to 15 sessions of CBT were offered. The job support adhered to the principles of IPS	Treatment as usual delivered by the general practitioner, the Norwegian Labour and Welfare Administration or other health professionals. Participants received a letter with information and encouragement to use available services and self-help resources One year after inclusion the control group was allowed to take part in the intervention	Study participants included in Reme 2015 Results adjusted for all measured confounders
(Reme et al. 2015) Norway	At Work and Coping Work-directed, individual cognitive behavioural therapy (CBT) combined with job support, delivered by teams of therapists and employment specialists at each site. Up to 15 sessions of CBT were offered. The job support adhered to the principles of IPS	Treatment as usual delivered by the general practitioner, the Norwegian Labour and Welfare Administration or other health professionals. Participants received a letter with information and encouragement to use available services and self-help resources One year after inclusion the control group was allowed to take part in the intervention	Study participants included in Overland 2018
(Salomonsson et al. 2017) Sweden	Cognitive behaviour therapy (CBT) Treatments were based on available evidence-based CBT protocols for each specific disorder. Depending on psychiatric disorder the length of CBT varied between 8 and 20 weekly sessions Return to work intervention (RTW-I) The treatment consisted of four central modules: (1) conceptualization, (2) psychoeducation, (3) planning and (4) monitoring. These modules were worked through in 10 sessions over a period of 20 weeks, initially weekly then follow-ups more sparsely Combination (COMBO) of CBT and RTW-I starting with three RTW-I sessions (the first three modules), followed by CBT for the specific disorder. Depending on the specific disorder and CBT protocol, the COMBO treatment thus varied between 10 and 25 sessions during a period of maximum 25 weeks	–	Study participants included in Salomonsson 2020 [14]
(Salomonsson et al. 2020) Sweden	Cognitive behaviour therapy (CBT) Treatments were based on available evidence-based CBT protocols for each specific disorder. Depending on psychiatric disorder the length of CBT varied between 8 and 20 weekly sessions Return to work intervention (RTW-I) The treatment consisted of four central modules: (1) conceptualization, (2) psychoeducation, (3) planning and (4) monitoring. These modules were worked through in 10 sessions over a period of 20 weeks, initially weekly then follow-ups more sparsely Combination (COMBO) of CBT and RTW-I starting with three RTW-I sessions (the first three modules), followed by CBT for the specific disorder. Depending on the specific disorder and CBT protocol, the COMBO treatment thus varied between 10 and 25 sessions during a period of maximum 25 weeks	–	Study participants included in Salomonsson 2017 [34] Subgroup analysis of the effects of the interventions separately for the Stress subgroup and the depression, anxiety, insomnia subgroup

### Three types of work-directed interventions

Based on our predefined description of work-directed interventions and in accordance with the interventions identified, the following intervention categories were defined:

**Individual Placement and Support (IPS)** is an employment support approach originally developed for severe mental disorders (e.g., psychosis, bipolar disorder). It involves supported job searching, paid placement, and on-the-job support for both the employee and employer. IPS has been adapted for mood and anxiety disorders as IPS-MA and Individual Enabling and Support (IES).

**Work-focused behavioral therapy** combines behavioral therapy techniques like Cognitive Behavioral Therapy (CBT) or Acceptance and Commitment Therapy (ACT) with a focus on treating symptoms related to physical and/or psychological health issues and improving quality of life. Recent innovations include integrating a work-focused approach involving meetings between the patient, the supervisor, and therapist to address ability to work and to facilitate RTW.

**Work-focused team-based support** utilizes a multidisciplinary team comprising health care professionals (physicians, psychologists, registered nurses) and an RTW-coordinator specialized in rehabilitation and return-to-work. The team identifies the patient’s resources and barriers to the RTW process, providing support through a participatory approach, which involves stepwise meetings with the patient, the supervisor, and healthcare representatives.

### Individual placement and support—summary of findings

Two studies, one from Denmark (Hellström et al. 2017) and one from Sweden (Bejerholm et al. 2017) evaluated the effects of IPS. The interventions analyzed in the studies varied somewhat and commonly dealt with coordination of stakeholder involvement (healthcare services, Public Employment Agency, Social Insurance Agency), counselling and on the job-training. The outcomes were competitive employment or education (Hellström et al. 2017) or employment rate (Bejerholm et al. 2017) measured at 12 (Bejerholm et al. 2017; Hellström et al. 2017) or 24 months (Hellström et al. 2017). A summary of the findings is presented in Table 3.

**Table 3:** Summary of findings and certainty of evidence of Individual placement and support, work-focused behavioral therapy, work-focused team-based support

**Population:** Persons with common mental disorder **Intervention:** Individual placement and support (IPS) **Countries:** Denmark, Sweden **Comparison:** Service as usual/traditional vocational rehabilitation					
Outcome	Number of participants (number of studies) Reference	Effect Oddsratio (OR), confidence interval (CI), percent (%), mean difference (MD), standard error (SE), within-group-difference, correlation (r), percent (%), standardized mean difference (SMD), hazard ratio (HZ), Cohens d (d), mean (m), standard deviation (sd))	Quality of the evidence (GRADE)	Reason/s for downgrading	Comments
Return to work	N = 386 (2) (Bejerholm et al. 2017; Hellström et al. 2017)	OR (95% CI) 1,52 (0,97–2,36) Employed % **IPS-MA** Intervention 44,2 Control = 38,7 **IES** Intervention = 42,2 Control = 4,0	Very low	Precision −2^a^ Indirectness −1^f^	The effect of IPS on return to work could not be assessed
Depression	N = 285 (2) (Bejerholm et al. 2017; Hellström et al. 2017)	MD = 0,27, SE = 0,45, p = 0,23 (HDIS, range 0–26) within-group-difference = z = 2,14, p = 0,03 (MADRS-S, range 0–54)	Very low	Bias −2^b, c^ Precision −1^a^	The effect of IPS on reported depression could not be assessed HDIS = Hamilton Depression 6-Item Scale MADRS-S = Montgomery Åsberg Depression Self Rate Scale
Anxiety	N = 229 (1) (Bejerholm et al. 2017)	MD = 0,66, SE = 0,48, p = 0,13 (HAIS, range 0–26)	Very low	Bias −1^b, c^ Indirectness −1^e^	The effect of IPS on reported anxiety could not be assessed HDAS = Hamilton Anxiety 6-Item Scale
Quality of life	N = 56 (1) (Hellström et al. 2017)	within-group-difference Intervention: z = −3,19, p = 0,001 Control: z = −1,05, p = 0,239 (MANSA, range 12–84)	Very low	Bias −2^c, d^ Indirectness −1^e^	The effect of IPS on reported quality of life could not be assessed MANSA = Manchester Short Assessment of quality of life

**Population:** Persons with common mental disorder **Intervention:** Work-focused behavioral therapy **Countries:** Denmark, Norway, Sweden **Comparison:** Standard care					
Outcome	Number of participants (number of studies) Reference	Effect Correlation (r), confidence interval (CI), percent (%), mean difference (MD), standardized mean difference (SMD), standard error (SE), hazard ratio (HZ), Cohens d (d), mean (m), standard deviation (sd))	Quality of the evidence (GRADE)	Reason/s for downgrading	Comments
Return to work	N = 1 356 (2) (Dalgaard et al. 2017a; Reme et al. 2015)	6.2%-units increase in work or worked time (r = 0,062, CI = 0,005–0,118) For sickness absence > 1 year: 7,4%-units increase (r = 0,074, CI = 0,011–0,370) HZ = 1,44, CI = 0,92–2,21	Low	Precision −2^a, d, g^	For persons on sickleave > 12 months the effect is larger
Sickness absence	N = 563 (2) (Finnes et al. 2019b; Salomonsson et al. 2017)	Number of sickness absence days **Combined CBT:** MD = 18, CI = 15,8–52 **CBT**: MD = 27, CI = 8,7–62,8 **ACT:** m = 19,4, sd = 27,7 **Usual care**: m = 17,4, sd = 27,7	Very low	Inconsistency −2^h^^, j^ Precision −1^a^	The effect of work-focused behavioral therapy on sickness absence could not be assessed
Income	N = 1 193 (1) (Overland et al. 2018)	MD = 12 148 NOK, SE = 12 780 For sickleave > 1 year: MD = 37 859 NOK, SE = 19 132	Very low	Precision −2^i^ Indirectness −1^e^	The effect of work-focused behavioral therapy on income could not be assessed NOK = Norwegian crowns
Depression	N = 936 (2) (Finnes et al. 2019b; Reme et al. 2015)	MD = −1,05, CI = −1,69 to −0,41 (HADS range 0–21)	Low	Bias −1^j,k^ Precision −1^a^	Work-focused behavioral therapy results in lower degree of reported depression after 12 months, compared to usual care HADS = Hospital Anxiety Depression Scale
Anxiety	N = 936 (2) (Finnes et al. 2019b; Reme et al. 2015)	MD = −0,50, CI = −1,32 to 0,33 (HADS range 0–21)	Very low	Bias −1^j,k^ Inconsistency −1^h^ Precision −1^a^	The effect of work-focused behavioral therapy on reported anxiety could not be assessed HADS = Hospital Anxiety Depression Scale
Quality of life	N = 925 (2) (Finnes et al. 2019b; Reme et al. 2015)	SMD = 0,11, CI = −0,03 to 0,25 (EQ-5D range 0–100, SWLS range 5–35)	Very lov	Bias −1 ^j, k^ Precision −2^a, f^	The effect of work-focused behavioral therapy on reported quality of life could not be assessed EQ-5D = The EuroQOL five dimensions questionnaire SWLS = Satisfaction with Life Scale
Stress	N = 136 (1) (Dalgaard et al. 2017a)	MD = −1,47, CI = −3,91 to 0,97 (PSS-10 range 0–40)	Very low	Precision −2^a,d^ Indirectness −1^e^	The effect of work-focused behavioral therapy on reported stress could not be assessed PSS-10 = Perceived Stress scale
Sleep	N = 136 (1) Dalgaard et al. 2017a) (2017)	MD = −0,54, CI = −2,24 to 1,15 (BNSQ range 14–70)	Very low	Precision −2^a, d^ Indirectness −1^e^	The effect of work-focused behavioral therapy on reported sleep could not be assessed BNSQ = Basic Nordic Sleep Questionnaire
Exhaustion	N = 100 (1) (Salomonsson et al. 2020)	d = −0,31, CI = −0,78 to 0,17 (SMBQ-22, range 1–6)	Very low	Precision -2^a, d^ Indirectness −1^e^	The effect of work-focused behavioral therapy on reported exhaustion could not be assessed SMBQ-22 = Shirom-Melamed Burnout Questionnaire
Work ability	N = 296 (1) (Finnes et al. 2019b)	ACT: m = 32,4, sd = 8,3 Usual care: m = 32,4, sd = 8,6 (WAI, range 7–49)	Very low	Precision −2^a, d^ Indirectness −1^f^	The effect of work-focused behavioral therapy on reported work ability could not be assessed WAI = The Work Ability Index

**Population:** Persons with common mental disorder **Intervention:** Work-focused team-based support **Countries:** Denmark, The Netherlands **Comparison:** Standard care					
Outcome	Number of participants (number of studies) Reference	Effect Odds ratio (OR), confidence interval (CI), mean difference (MD), hazard ratio (HZ))	Quality of the evidence (GRADE)	Reason/s for downgrading	Comments
Return to work	N = 595 (2) (Hoff et al. 2022; Lammerts et al. 2016)	Entrance or return to work *Netherlands*: HZ = 1,15, CI = 0,61–2,16 *Denmark*: HZ = 0,96, CI = 0,71–1,29 OR = 0,64, CI = 0,39–1,05	Low	Bias, −1^g^ Precision, −1^a^	Work-focused team-based support results in increased entrance or return to work after 12 months, compared to usual care
Depression	N = 533 (2) (Hoff et al. 2022; Lammerts et al. 2016)	MD = −0,58, CI = −1,11 to −0,04 (FDQ: range 0–12)	Low	Bias −1^j^ Precision −1^a^	Work-focused team-based support results in lower degree of reported depression after 12 months, compared to usual care FDQ = Four Dimensional symptom Questionnaire
Anxiety	N = 402 (2) (Hoff et al. 2022; Lammerts et al. 2016)	MD = −0,29, CI = −1,25 to 0,68 (FDQ: range 0–12)	Very low	Bias −1^j^ Precision −2^a, f^	The effect of work-focused team-based support on reported anxiety could not be assessed FDQ = Four Dimensional symptom Questionnaire
Stress	N = 278 (1) (Hoff et al. 2022)	MD = 0,58, 98,3 CI = −0,62 to 1,77 (PSS range 0–40)	Very low	Precision −2^a^ Indirectness −1^e^	The effect of work-focused team-based support on reported stress could not be assessed PSS = Perceived Stress Scale
Quality of life	N = 402 (2) (Hoff et al. 2022; Lammerts et al. 2016)	*Netherlands* Physical: MD = −2,20, CI = −5,90 to 1,50 Mental: MD = 2,50, CI = −2,15 till 7,15 (SF 36, range 0–100) *Denmark* MD = 0,01, CI = −0,05 to 0,03 (EQ5-D, range 0–100)	Very low	Bias −1^j^ Precision −2^a, f^	The effect of work-focused team-based support on reported quality of life could not be assessed SF-36 = Short Form Health Survey EQ-5D = The EuroQOL five dimensions questionnaire
Exhaustion	N = 278 (1) (Hoff et al. 2022)	MD = 2,07, 98,3% KI = −2,09 to 6,24 (KEDS, range 0–47)	Very low	Precision −2^a^ Indirectness −1^e^	The effect of work-focused team-based support on reported exhaustion could not be assessed KEDS = Karolinska Exhaustion Disorder Scale
Work ability	N = 278 (1) (Hoff et al. 2022)	MD = 0,18, 98,3% CI = −1,46 to 1,82 (WSAS, range 0–40)	Very low	Precision −2^a^ Indirectness −1^e^	The effect of work-focused team-based support on reported work ability could not be assessed WSAS = Work and Social Adjustment Scale

^a^Wide confidence interval

^b^Low reseponse rate

^c^Incomplete reported data (within-group-analysis)

^d^Few participants

^e^One study

^f^No significant effect

^g^Hazard ratio

^h^Results in different directions

^i^Large standard error

^j^Missing data

^k^Heterogenous interventions

Hellström et al. (Hellström et al. 2017) evaluated IPS modified for people with mood and anxiety disorders (IPS-MA) compared to standard service containing: support from ‘job centers’ including for example courses, counselling, or job-training. The number and length of meetings were adjusted to the individual’s needs.

Bejerholm et al. (Bejerholm et al. 2017) compared Individual Enabling Support (IES) following the principles of IPS, with traditional vocational rehabilitation. The intervention lasted for 12 months. The support was based on motivational interviewing and cognitive strategies and was provided in accordance with ten IES principles, e.g., development of motivational and cognitive strategies, and competitive employment as a primary goal. The control group received traditional vocational rehabilitation delivered by different professionals and organizations.

### Effects on return to work

**Fig. 2:**
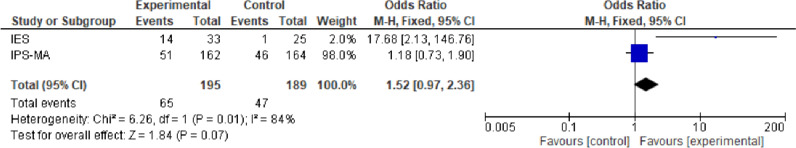
The effect of individual placement and support on number of persons returning to work or education, compared to standard care, 12 months follow-up, odds ratio (Bejerholm et al. 2017; Hellström et al. 2017)

The results show that at the 12-month follow-up, IES had a positive effect on the employment rate (Bejerholm et al. 2017). Hellström et al. reported that compoared to standard care, IPS-MA had no significant effects on competitive employment or education (Hellström et al. 2017) when compared to care as usual. Overall, the meta-analysis showed no significant differences from standard care (Fig. 2). However, the effect of Individual Placement and Support on return to work could not be assessed, mainly due to large confidence intervals (Table 3).

### Effects on self-reported depression, anxiety, and quality of life

The effect of Individual Placement and Support on depression, anxiety, and quality of life could not be assessed, mainly due to the low response rate and incomplete data (Table 3).

### Work-focused behavioral therapy—summary of findings

Seven articles, from Denmark, Norway and Sweden, reported the results of three studies evaluating the effects of CBT (Dalgaard et al. 2017a, 2017b; Overland et al. 2018; Reme et al. 2015; Salomonsson et al. 2017; Salomonsson et al. 2020) and one study evaluating the effects of Acceptance and Commitment Therapy (ACT) (Finnes et al. 2019b). A summary of the findings is presented in Table 3.

Overland et al. (Overland et al. 2018) and Reme et al. (Reme et al. 2015) compared work-focused CBT in combination with individual job support from the Norwegian Labor and Welfare Administration or other stakeholders (e.g., healthcare services). About 40% of the participants were on full-time sick leave, about 15% on part-time sick leave and 10% were unemployed. All participants in the intervention group received up to 15 sessions of CBT, and of those, 32% received individual support based on IPS principles.

Salomonsson et al. (Salomonsson et al. 2017, 2020) compared CBT, including an RTW-intervention with CBT. The number of sessions in the intervention- and control groups varied depending on the psychiatric disorder. A majority of the participants had symptoms of exhaustion and were absent due to sickness (duration from one up to six months part- or full-time).

Dalgaard et al. (Dalgaard et al. 2017a, 2017b) compared work-focused CBT, with a control group receiving clinical examination. The intervention included meetings at the workplace for discussion about modifications, for example workload or professional roles.

Finnes et al. (Finnes et al. 2019b) compared ACT, including a convergence dialogue, with standard care provided by primary healthcare or social services. The aim of the convergence dialogue with the workplace was to reach agreement about long- and short-term solutions in relation to RTW.

### Effects on return to work

Compared to standard care or no intervention, work-focused CBT was shown to increase work participation by 6.2% units (Reme et al. 2015) after 12 months and also increase the probability of RTW after 44 weeks (Dalgaard et al. 2017a) (Table 3). Because the reported data were incomplete, it was not possible to undertake a meta-analysis.

The effect on absence due to sickness and income could not be assessed, mainly due to contradictory results and large confidence intervals or standard error (Table 3). Overall, it is possible that work-focused CBT may have a positive effect on RTW at 12 months follow up. A greater effect was observed for those on sick leave for longer than 1 year.

### Effects on self-reported depression, anxiety and quality of life

**Fig. 3:**
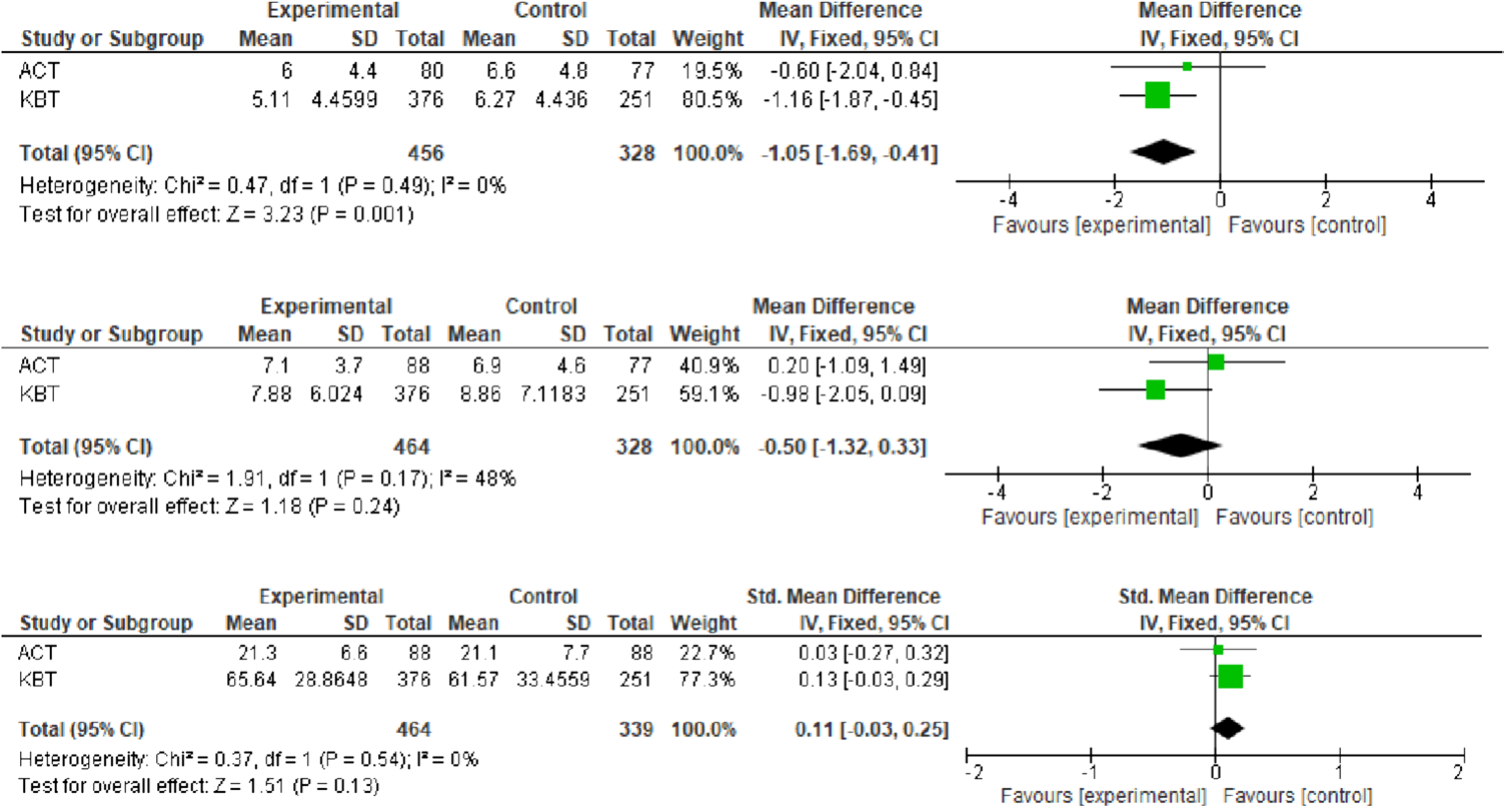
Effect of work-focused behavioral therapy on **a**: depressive symptoms measured with Hospital Anxiety and Depression Scale, 9 (Finnes et al. 2019b) and 12 months follow-up (Reme et al. 2015); **b**: anxiety symptoms measured with Hospital Anxiety and Depression Scale, 9 (Finnes et al. 2019b) and 12 months follow-up (Reme et al. 2015); **c**: on quality of life measured with the EuroQOL five dimensions questionnaire (Reme et al. 2015) or Satisfaction with Life Scale (Finnes et al. 2019b; Reme et al. 2015). Comparisons received standard care

Reme et al. and Finnes et al. measured symptoms of depression and anxiety using Hospital Anxiety Depression Scale (HADS). Work-directed CBT resulted in a decrease in depressive symptoms at the 12-month follow-up (Reme et al. 2015) and a corresponding effect of ACT at the 9-month follow-up (Finnes et al. 2019b) (Table 3). The interventions were adequately consistent and were therefore included in a meta-analysis. This showed that compared to standard care, work-focused behavioral therapy resulted in a reduction of depressive symptoms (Fig. 3). However, the effect of the interventions on anxiety was inconsistent (Fig. 3). Hence, no conclusions can be drawn about the effects of the interventions on the symptoms of anxiety compared to standard care. Overall, it is possible that work-focused behavioral therapy may reduce symptoms of depression at the 12-month follow-up. However, this effect should be interpreted with caution, as the level of depression is within the range of no depression. Thus, although this result shows a minor effect in terms of reduced symptoms of depression, it would appear to be of little clinical relevance. With respect to the effect on anxiety, the meta-analysis shows inconsistent results, with large confidence intervals. Hence, the effect of work-focused behavioral therapy on symptoms of anxiety could not be assessed.

Furthermore, Reme et al. and Finnes et al. reported measurements of quality of life. Neither work-directed CBT at the 12-month follow-up (Reme et al. 2015), or ACT at the 9 months follow-up (Finnes et al. 2019b) had a significant effect on self-reported quality of life (Table 3). The meta-analysis indicated that compared to standard care, the interventions resulted in a minor, although not statistically significant, increase in quality of life compared to standard care (Fig. 3). Hence, the effect of work-focused behavioral therapy on reported quality of life could not be assessed, mainly due to a large confidence interval.

### Work-focused team-based support—summary of findings

Two studies, from The Netherlands (Lammerts et al. 2016) and Denmark (Hoff et al. 2022) evaluated the effects of work-focused team-based support. A summary of the findings is presented in Table 3.

Lammerts et al. (Lammerts et al. 2016) compared a standardized form of occupational healthcare early after sick leave, with standard care provided by the Dutch Social Security Agency. Hoff et al. (Hoff et al. 2022) evaluated integrated vocational rehabilitation and mental health care, in addition to standard care, and compared this to standard care alone.

### Effects on return to work

The interventions were sufficiently consistent, but the data were inadequately reported (only hazard ratios) (Lammerts et al. 2016). Hence, no meta-analysis was conducted. No effects on the number of weeks until RTW were reported from the two studies. However, in the Danish study, 56% returned to work after 12 months, compared with 46% in the control group. Thus, a narrative synthesis indicates that compared to standard care, work-focused team-based support may increase RTW (Table 3).

### Effects on self-reported depression and anxiety

**Fig. 4:**
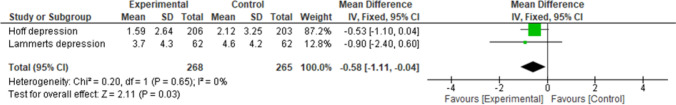
Effect of work-focused team-based support on depression compared to standard care at 12 months follow-up (Hoff et al. 2022; Lammerts et al. 2016)

Lammerts et al. (Lammerts et al. 2016) and Hoff et al. (Hoff et al. 2022) included self-reported symptoms of depression and anxiety, measured by the Four Dimensional Symptom Questionnaire and compared this with standard care at the 12-month follow-up (Hoff et al. 2022; Lammerts et al. 2016) (Table 3). The meta-analysis revealed a statistically significant reduction in depression associated with work-focused team-based support (Fig. 4). However, the effect should be interpreted with caution, as the levels of depression are within the range of no depression.

Overall, the effect of work-focused team-based support on symptoms of anxiety, stress, exhaustion, quality of life, and work ability, could not be assessed, mainly due to the wide confidence intervals and low response rate (Table 3).

### Ethical aspects

Several ethical aspects were identified. Work-directed interventions might increase the individual’s feeling of guilt and shame: to a large extent the interventions focus on and are directed towards the individual’s mental health, rather than, for example, problems in the workplace. Further, work-directed interventions might affect the individual autonomy. Thus, pointing to questions whether participation was voluntary, whether an individual had the opportunity to control and select some details of the interventions. The issue of whether CMD-symptoms might be an obstacle to making informed and autonomous decisions must be considered. The work-directed interventions might affect personal integrity and the individual’s control over the flow of personal information. For example, work-focused team-based support included meetings between the patient, healthcare representatives and employer representatives. In this context, it might be difficult for the participant to withhold personal and health-related information which they did not wish to share with the employer.

## Discussion

Our review concludes that interventions involving the workplace could potentially increase the probability of returning to work. The studies on IPS with the workplace involved had a very low certainty of evidence, making it impossible to assess the impact of these interventions. A very low certainty of evidence, however, does not necessarily mean that there is no effect: it highlights the need for more well-designed studies of this topic. Studies of behavioural therapy and team-based support yielded low certainty of evidence, which implies that it is possible that future research might change these results.

Our results are largely consistent with previous systematic reviews targeting people with CMDs (Finnes et al. 2019a; Joyce et al. 2016; Nigatu et al. 2016), mental health conditions (Fadyl et al. 2020), mental disorders (Dewa et al. 2015), depression (Nieuwenhuijsen et al. 2020) or adjustment disorders (Arends et al. 2012): there is no convincing evidence that the interventions involving the workplace led to return to. As in our review, previous systematic reviews have included a range of interventions (e.g., targeting the individual’s workability, RTW behaviour, coping strategies, problem-solving skills, and interpersonal behaviours or organizational change). Besides the variety across the interventions, these are based on different mechanisms. As suggested by Nieuwenhuijsen and colleagues (Nieuwenhuijsen et al. 2020) the mechanisms could be broadly classified into (a) improving working conditions for supporting the employee to overcome the barriers for returning to work, e.g., by the adjustment of working hours or work tasks, or (b) the improvement of depressive or other psychological symptoms using medication and/or therapy (e.g., CBT) (Nieuwenhuijsen et al. 2020). In our systematic review, the included interventions were grouped into three categories. In addition, the interventions included in the categories ‘IPS’ and ‘Work-focused team-base support’ were mainly based on the first mechanism, while the interventions in the category ‘Work-focused behavioural therapy’ utilized a combination (Nieuwenhuijsen et al. 2020). However, irrespective of clarity of mechanisms, we cannot draw any firm conclusions regarding the interventions’ effectiveness. Another problem in the categorization of interventions is the potential overlap between the interventions. Our categorization was based on the main content of each intervention. However, in the study by (Reme et al. 2015) an intervention with CBT and individual support based on IPS-principles was evaluated. All participants in the intervention group received up to 15 CBT sessions, and of those, 32% received individual support based on IPS-principles. We have based our categorization on the fact that CBT was delivered to all study participants in the intervention group and a lesser amount receiving individual support. Still, with the range of intervention and the need of exploring why an intervention results in the desired effect or not, we suggest a thorough examination of the adherence to an intervention’s components by e.g., evaluating the reach, dose delivered, received and underlying mechanisms. This could be done by conducting a process evaluation in parallel to an effectiveness trial. A process evaluation could add to the current knowledgebase by informing results from a randomized controlled trial with how much of each component that needs to be delivered, the uptake of the intervention and components and the users’ perceptions about barriers to, and facilitators of the intervention. Having the work-directed intervention in mind, i.e., a complex intervention commonly delivered and evaluated at individual, organizational societal levels (Skivington et al. 2021) a process evaluation could develop the interpretation of the results from an effectiveness trial. For example, Arends and colleagues’ (Arends et al. 2012) reported that several of the intervention’s components (e.g., inventory of problems and/or opportunities and support needed) were linked to the outcome recurrent sickness absence. Hence, their process evaluation provided an example of how a thorough examination of an intervention’s components explain the intervention’s effectiveness.

Even if our systematic review did not include studies with a qualitative design, the addition of study participants’ perspective of the interventions could provide sufficient knowledge to our findings. Previous studies reporting the individuals’ experiences of participating in work-directed intervention have shown that the individual’s learned from receiving individual support in their preparation of RTW. The intervention by Wisenthal and colleagues contained a mapping of work ability, need and motivation for RTW, which contributed to the participants’ self-reflection, visualizing their resources and clarifying demands (Wisenthal et al. 2019). Further, the professionals providing work-directed interventions needed an including attitude regarding the individual’s situation and experiences in combination with their medical expertise (Andersen et al. 2014; Strömbäck et al. 2020). However, besides the support needed when preparing the RTW, support is also needed during the RTW to achieve a seamless transition from sickness absence into re-entering work (Wästberg et al. 2013). Among non-employed individuals with long-term conditions, support is needed throughout the process of gaining a paid employment, e.g., by sufficient collaboration with the involved stakeholders (Fadyl et al. 2022). These findings add to the results of our systematic review by, on the one hand, using interventions which support the development of self-efficacy and motivation. On the other hand, the participants asked for more ‘hands on-support’ during and after they had returned to work. We conclude that the interventions included in our systematic review could benefit from being adjusted to individual needs of behavioural change and support.

In addition to previous systematic reviews, our review highlights several ethical aspects arising from work-directed interventions. The included interventions suggest increased cooperation between stakeholders, e.g., the individual on sickness absence, his/her employer, health care- and Social Insurance Agency’s representatives. Our ethical analysis indicates that the explored interventions may affect the individual’s autonomy, personal integrity and control over the sharing of sensitive information. These results are in line with Holmlund et al. (Holmlund et al. 2023). In addition, Holmlund and colleagues revealed that unclear roles among the professionals involved in delivering work-directed interventions implied unequal access to support (Holmlund et al. 2023). Another ethical analysis of a similar intervention showed ethical challenges due to conflicting goals on organizational and individual levels, e.g., the intervention challenged organizational values on fairness and justice, and introduced a need for the individual to juggle the roles of an employee and a patient (Karlsson et al. 2024). The interventions investigated in our systematic review presume a common goal of reintegrating the employee back to work, among the involved stakeholders. However, our results show that work-directed interventions come with ethical ‘costs’ on behalf of (first, and foremost) the individual, but—as shown by previous studies (Holmlund et al. 2023; Karlsson et al. 2024) on the behalf of the involved stakeholders and organizations. Given the inconclusive results shown by our, and previous systematic reviews of work-directed interventions, the results from our ethical analysis should be taken into consideration when planning and conducting work-directed interventions. Further, these results might guide policy- and decisionmakers whether to implement work-directed interventions.

### Allowing for quasi-experimental designs in systematic reviews of effectiveness

Despite our search of quasi-experimental designs, we did not find any studies which met the inclusion criteria. Although quasi-experimental studies examining RTW outcomes for sick leave individuals exist, they encompass broader diagnostic populations beyond Common Mental Disorders (CMD), thus being excluded from our review. For instance, Hägglund et al. (Hägglund et al. 2020) analysed the impact of CBT on individuals with mild or moderate mental illness, and Hägglund (Hägglund 2013) assessed the effects of stricter enforcement of eligibility criteria in the Swedish sickness insurance system. These studies belong primarily to the field of economics, highlighting a discrepancy in population focus across research disciplines.

However, we suggest future systematic reviews to allow the inclusion of quasi-experimental designs when evaluating an intervention’s effectiveness, as these may often be considered to have a high external validity. Quasi-experimental designs offer a valuable alternative when ethical or logistical considerations prevent the implementation of true experiments. While randomised control trials aim to establish causal effects through random assignment, quasi-experimental designs achieve a similar goal without relying on true randomization. Instead, subjects are grouped based on predetermined criteria, mirroring random assignment to mitigate individual selection biases common in non-randomized experiments. Key quasi-experimental methods include Regression Discontinuity, Differences-in-Differences, and the Instrumental Variable method, as detailed, for instance, by Angrist and Pischke (Angrist and Pischke 2009).

Further, quasi-experiments often present several advantages. These include typically larger sample sizes, a reduced risk of biased population sampling, and the absence of issues related to participants and/or caseworkers being aware that they are part of a study. Quasi-experiments may have these advantages because they involve real-world interventions that have already been implemented without the explicit purpose of evaluation. Consequently, the concern that participants are aware of being part of a study is not an issue. Moreover, their use of retrospective register data helps alleviate problems associated with small sample sizes and attrition.

Quasi-experiments also have shortcomings, particularly if the fundamental assumption for identifying a causal treatment effect is unlikely to be met. Comparing the advantages and disadvantages of experiments versus quasi-experiments is not straightforward, as it hinges on the quality and context of the specific study. Our rationale for including quasi-experimental studies in the review lies in their potential to furnish evidence as compelling as RCT studies, underscoring their significance in systematic reviews.

Exploiting the potential of quasi-experiments to study subpopulations of interest, such as CMD, within larger sample sizes could contribute to improving research quality. Furthermore, quasi-experimental methods can be utilized for evaluating existing interventions and can be implemented gradually in different regions to leverage temporal variations for evaluation purposes. It is of interest to note that there are also RCT studies conducted in economics that evaluate labor market interventions but also include broader populations than those with CMD, such as the studies by Fogelgren et al. (Fogelgren et al. 2023), Engström et al. (Engström et al. 2017) and Laun and Skogman Thoursie (Laun and Skogman Thoursie 2014).

### More studies of high scientific quality are needed

A recurring conclusion from the previous reviews is that more studies of high scientific quality are needed. We agree with this conclusion. About half the studies meeting our inclusion criteria were excluded from the review because they were assessed as having a high risk of bias. We have identified the following methodological aspects for consideration in future research.

Firstly, a recurrent problem is the small number of participants and underpowered trials. Experiments are resource-intensive, and the cost of large-scale experiments is significant. This means that RCT studies often become small-scale. Most studies included in our review report recruitment difficulties, which implies a risk that the pre-estimated group size cannot be achieved. In addition, many studies are conducted at a few local offices or centres where the participants are not necessarily representative of a broader population. These aspects reduce the external validity.

Secondly, it is difficult to withhold information that the participants are part of a study. Consent from participants is usually required and neither the participants nor those providing the interventions are blinded. While initially it may be feasible to withhold information regarding the assigned intervention from individuals, it is important to consider that the intervention unfolds over a specific duration, and participants can hardly be shielded from external information indefinitely. These aspects reduce the internal validity.

Thirdly, the studies lack detailed descriptions of the content of interventions, comparisons groups and so-called ‘co-interventions’. With reference to ‘care as usual’ only two studies reported the use of drug treatment (Dalgaard et al. 2017a, 2017b; Salomonsson et al. 2017, 2020). Such treatment is commonly used for reducing symptoms of e.g., anxiety and/or depression and could possibly influence the outcome of sickness absence. The absence of such information makes it difficult to interpret the results of the studies and limits the ability to replicate them.

Fourthly, in line with recent research findings, we support the necessity to establish standardized outcome measures, a ‘Core Outcome Set’ (see Hoving et al. 2018; Ravinskaya et al. 2023; Ravinskaya et al. 2022). The main argument is to be able to compare studies. For example, while some studies focus on the duration until return to work, others emphasize the share of individuals who have returned to work, or the duration of sick leave. Nonetheless, our study reveals additional crucial insights regarding a ‘Core Outcome Set’. First, it is important to have a common approach how to calculate outcomes. Even if the same outcome information is available, some authors favour odds ratios, while others prefer alternative measure such as the share returned to work. Secondly, we emphasize the merits of utilizing core outcomes derived from register data. We argued for the inclusion of quasi-experimental studies in our report, where register data is essential. Once again, the establishment of a ‘Core Outcome Set’ is paramount. The question is if this core set of outcomes can also include registry-based measures of health. This could involve, for example, the number of days in outpatient care, inpatient care, and the number of prescribed doses of medication.

Finally, our systematic review evaluated three distinct interventions, IPS and ‘Work-focused’ team-base support and ‘Work-focused’ CBT. As already argued, to explore why an intervention results in the desired effect or not, we advocate process evaluations in order to learn the underlying mechanisms for the potential success of an interventions. This also opens up the question whether an intervention could be more successful if it, for example, incorporated elements from both IPS and CBT. This suggest that studies do not only randomize individuals to singular treatment arms, such as IPS or CBT, but also to a combined treatment arm, such as IPS and CBT together.

### Methodological considerations

One strength of our study is the comprehensive literature search in international databases, citation searches, and different publication types, including ‘grey literature’. The risk of overlooking any significant studies is small. Further, the certainty of the quantitative results has been assessed by applying the international GRADE system, which means that a structured assessment was made of five domains.

With regard to limitations, our review included articles reporting RCTs conducted in Sweden, Denmark, Norway and The Netherlands. These countries have different social insurance systems which could potentially affect the outcome, and this should therefore be considered when interpreting the results. Another limitation is our categorization of the included interventions. Even if an intervention had a specific content, e.g., CBT, it was not possible to determine whether the content was the same across studies, not whether the competence and training of those implementing the intervention affected the outcome.
